# Supplementary material for: High-performance coherent optical modulators based on thin-film lithium niobate platform
Source: Nat Commun. 2020 Aug 6;11:3911. doi: 10.1038/s41467-020-17806-0 (PMC7411015; doi:10.1038/s41467-020-17806-0)
Supplement: Supplementary file 1 — Supplementary Information [file 41467_2020_17806_MOESM1_ESM.pdf]

Supplementary Information for:

Xu et al.

High-performance Coherent Optical Modulators based on Thin-film Lithium Niobate Platform

Supplementary Information

High-performance Coherent Optical Modulators based on Thin-film Lithium Niobate Platform

Mengyue Xu[1], Mingbo He[1], Hongguang Zhang[2,3], Jian Jian[1], Ying Pan[1], Xiaoyue Liu[1], Lifeng Chen[1], Xiangyu Meng[1], Hui Chen[1], Zhaohui Li[1], Xi Xiao[2,3], Shaohua Yu[2,3], Siyuan Yu[1], Xinlun Cai[1]

[1]State Key Laboratory of Optoelectronic Materials and Technologies and School of Electronics and Information Technology, Sun Yat-sen University, Guangzhou 510275, China.

[2]National Information Optoelectronics Innovation Centre, China Information and Communication Technologies Group Corporation (CICT), Wuhan 430074, Hubei, China

[3]State Key Laboratory of Optical Communication Technologies and Networks, China Information and Communication Technologies Group Corporation (CICT), Wuhan, 430074, China

Correspondence and requests for materials should be addressed to X.X and X.C

Supplementary Note 1 | Comparison between LNOI modulator and SOI-LNOI hybrid modulator

The monolithic LNOI device can be regard as the next generation of the widely used conventional lithium niobate modulators, which can find practical applications in near future. In contrast, SOI-LNOI hybrid device is a technology for longer term. Silicon photonics is becoming the leading technology for photonic integrated circuits. By harnessing the tool sets and process flows in CMOS foundries, the technology offers advantages of low-cost, high-volume and reliable manufacturing. Recently, thin-film LN is co-integrated on silicon photonics in a hybrid way, which opens up new avenues for high-performance silicon based optical modulators. This hybrid integration is promised to offer one solution for future photonic integrated circuits with high-level of integration, which combines the 'best-in-breed' components, including thin-film LN modulators, silicon passive devices and III-V active devices.

Supplementary Note 2 | LN MMI couplers and mode transition from single mode waveguide to 4-µm-wide LN waveguide

We employ 1 × 2 multimode interference (MMI) couplers to provide symmetric power splitting and combing in our modulators. The MMI couplers considered here are based on the etched LN waveguides with a slab thickness of 300 nm and a rib height of 300 nm. The length of the multimode region L_M was designed to be 64 µm, and the width of the multimode region W_M and the wider ends were set as 9.654 µm and 4.54 µm, respectively (Supplementary Figure 1a). Three identical 50-µm-long linear tapers are used as adiabatic tapers and connected to single-mode waveguides. Supplementary Figure 1b shows the simulated excess loss for different wavelengths.

To measure the excess loss of MMI coupler, we fabricated the MMI couplers arranged in a cascaded configuration, as depicted in Supplementary Figure 1c. Supplementary Figure 1d shows the measured transmission of the MMI couplers at the wavelength of 1550 nm. We extract the excess loss of ~ 0.054 dB from the slope of the fitting line. Next, we experimentally demonstrated the performance of our 3-dB MMI couplers by integrating two MMI couplers into an unbalanced MZI (Supplementary Figure 1e). Supplementary Figure 1f shows the static extinction ratio is greater than 40 dB, which indicates that the

power splitting ratio of the MMI is very close to 50:50.

In the practical devices, we adopt single-mode waveguides (width of 1 μm) for all the other parts of the device (including the access waveguides for MMI and all the bend waveguides) except the phase modulation parts. We use adiabatic waveguide tapers to connect 1-μm-wide single-mode waveguides and 4-μm-wide phase modulation waveguides, which does not incur any conversion from fundamental mode to high-order modes (Supplementary Figure 2a). In this way, we can make sure that only the fundamental mode is excited when the light propagates in the device (Supplementary Figure 2b). In Fig. S2c, the simulated result shows the designed adiabatic taper can achieve a transmission of approximately 99.9 %.

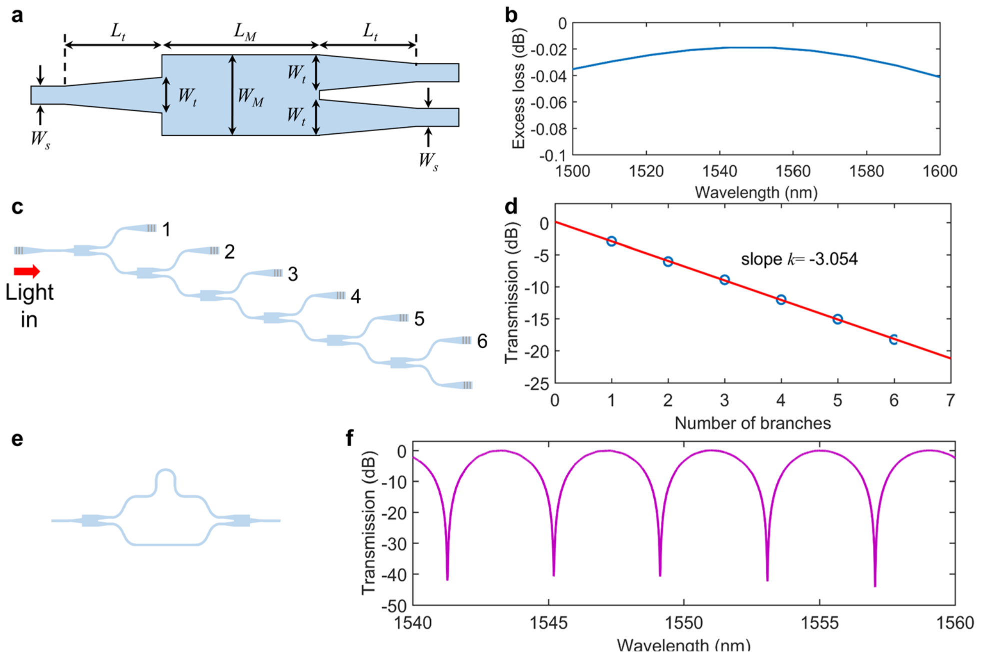

Supplementary Figure 1 | MMI performance. a Schematic of an MMI coupler. **b** Simulated excess loss of the MMI for different wavelengths. **c** Schematic of the MMI couplers arranged in a cascaded configuration for loss measurement. **d** Transmittance measured at of the output of the cascaded MMI couplers as a function of the number of branches (blue circle) a linear fit (red solid line). **e** Schematic of an unbalanced MZI with two 1 × 2 MMI couplers. f Measured static extinction ratio of the unbalanced MZI.

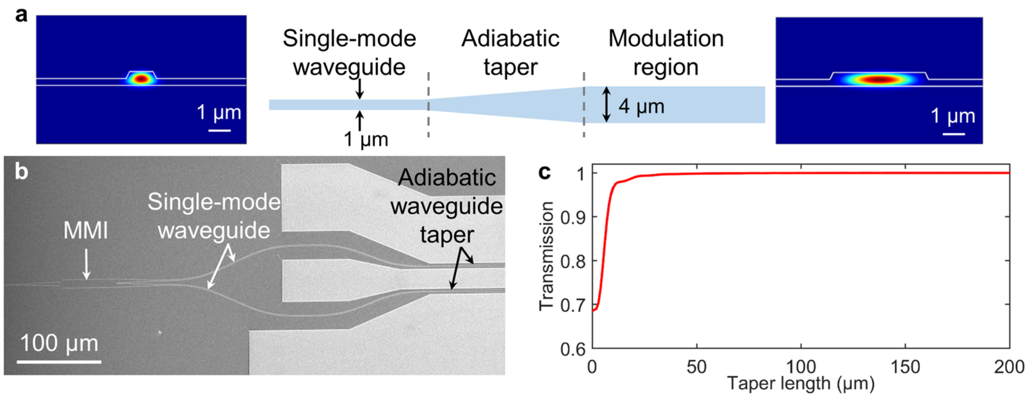

Supplementary Figure 2 | Mode transition. a Schematic of an adiabatic waveguide taper and mode profile of a 1-um-wide waveguide and a 4-um-wide waveguide, respectively. **b** SEM image of a MMI

and adiabatic tapers. **c** Simulated transmission of the adiabatic waveguide taper as a function of taper length.

Supplementary Note 3 | AFM measurements and loss analysis

The sidewall roughness plays the important role in inducing scattering loss. We optimise the lithography and etching process to reduce surface scattering from the sidewalls. The 3D AFM scan of the fabricated LN waveguide is shown in Supplementary Figure 3a. The sidewall angle of 64° and the etch depth of 300 nm are extracted from the line profile of the ridge waveguide (Supplementary Figure 3b). A 2D image of the sidewall surface is shown in Supplementary Figure 3c. The root-mean-squared (RMS) roughness of the sidewalls of the LN waveguides is 1.24 nm in a 2-by-0.05-μm AFM scan area.

There are two kinds of optical waveguides in our devices: 1-μm-wide single mode waveguide and 4-μm-wide waveguide. To investigate the propagation loss of these waveguide, we fabricated LN microrings with the 1-μm-wide waveguide and 4-μm-wide waveguide. The etching depth of the microring waveguide are the same as the LN waveguide used in our modulators. From the measured Q factors of the transmission spectra (Supplementary Figure 4a,c), we can obtain the propagation loss is 0.3 dB/cm (1-μm-wide waveguide) and 0.15 dB/cm (4-μm-wide waveguide), respectively.

To further confirm the additional loss from bias and modulation electrodes, we fabricated Titanium (Ti) electrodes along the microring with 1-μm-wide single mode waveguide and Au electrode along the microring with 4-μm-wide waveguide, respectively. The gaps between the electrodes and the waveguides were the same as those used in the modulator device. The Q factors were measured again for the microrings with electrodes (Supplementary Figure 4b,d), and the loss measured loss results indicates that the electrodes do not incur substantially additional absorption loss.

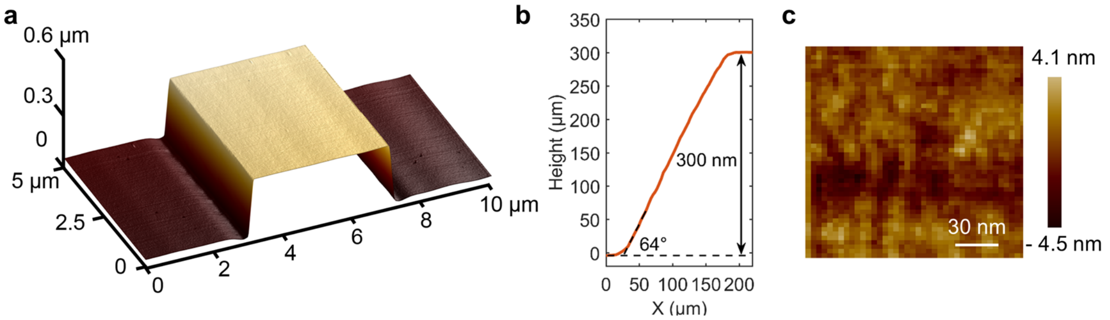

Supplementary Figure 3 | AFM measurement of LN waveguide. a 3D AFM scan of a 4-μm-wide LN waveguide. **b** AFM line profile of the sidewall of LN waveguide. **c** 2D image of the sidewall of LN waveguide.

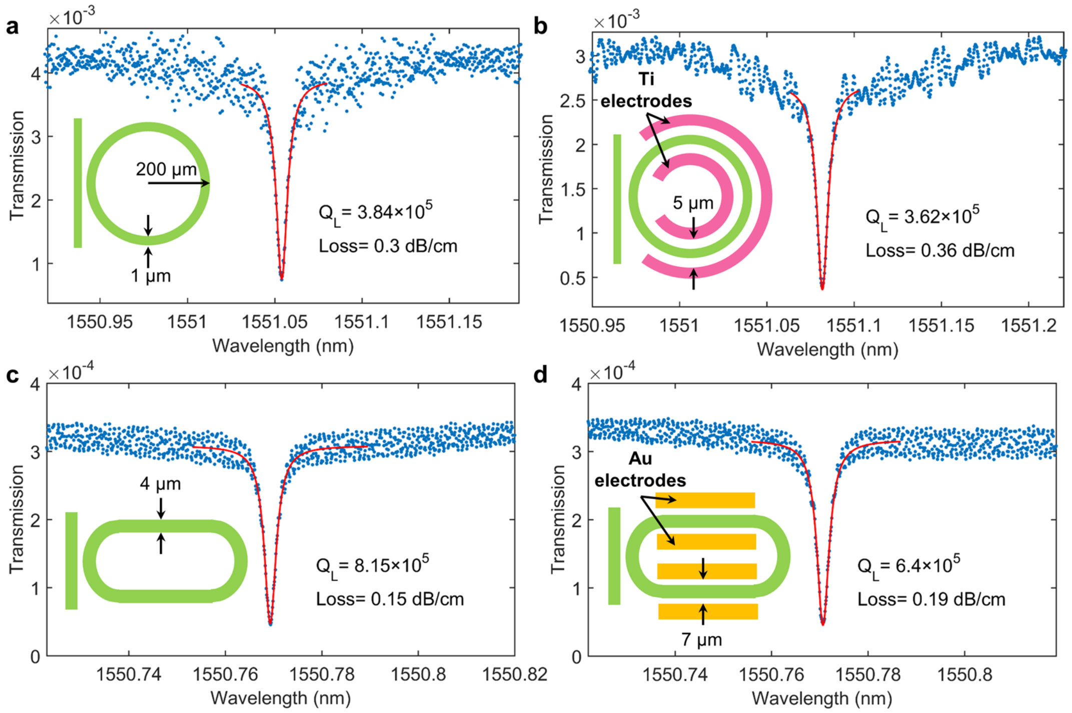

Supplementary Figure 4| Optical transmission spectra of the microring resonators. Measured transmission spectra (blue dots) of and Lorentz fittings (red lines) of **a** 1-μm-wide LN microring resonator, **b** 1-μm-wide LN microring resonator with Ti electrodes, **c** 4-μm-wide LN racetrack resonator and **d** 4-μm-wide LN racetrack resonator with Au electrodes.

Supplementary Note 4| Design of phase modulation waveguides and travelling-wave electrodes

It is essential for an electro-optic (EO) modulator to achieve a strong interaction between the optical mode and the actively modulated material in the modulation region. On the other hand, through designing the top width of the ridge LN waveguide (w), we can further enhance the overlap between the electrical and the optical fields. Reducing the electrodes spacing inflicts trade-offs between electro-optic bandwidth (BW), insertion loss (IL) and half-wave voltage (V_π). In our simulation, the electrodes spacing vary with the width of the waveguides to keep the same metal absorption loss (simulated at 0.04 dB cm^{-1}). In this section, we use the ratio BW/ V_π as a figure of merit to estimate the performance of LN modulator here.

In order to calculate the V_π, we first calculated the electric field distribution (E_z) in a commercial software (COMSOL Multiphysics). The applied voltage (1 V in our simulation) causes the refractive index change in LN material. Some of the structural parameters are defined as etch depth of 300 nm, LN film thickness of 600 nm and SiO_2 thickness of 4.7 μm. We then calculated the effective refractive index change (Δn_{eff}) of the fundamental TE mode at a wavelength of 1550 nm in an optical mode solver (Lumerical Mode solution). Finally, V_π of 1-cm push-pull Mach-Zehnder modulator can be calculated as:

$$V_\pi = \frac{1550\ \ nm}{2\Delta n_{eff} \cdot 1\ \ cm} \cdot 1V$$

Supplementary Figure 5**d** show the calculated V_π (on the left axis of) as a function of w at the given modulation length of 1 cm. The lowest V_π = 2.36 V is obtained around w= 2 μm. The corresponding

electrode spacing is 5.62 μm. This small electrode spacing increase the overlap between the optical mode and the electrical field. However, this simultaneously results in increasing the RF attenuation (α_m) and bringing difficulties in optimizing the performance of the transmission line.

The frequency response of travelling-wave modulator is dominated by the mismatch between RF effective mode index (n_m) and optical group index (n_o), the characteristic impedance (Z_c) and the RF attenuation (α_m) of the transmission line. We calculate this three main factors using ANAYS HFSS. The electro-optic response EO_{S21} of a travelling-wave modulator can be expressed as [S1]:

$$EO_{S21} = \left|\frac{Z_{in}}{Z_{in}+50}\frac{(50+Z_c)F(u_+)+(50-Z_c)F(u_-)}{(50+Z_c)e^{\gamma_m L}+(50-Z_c)e^{-\gamma_m L}}\right|^2$$

where, $Z_{in} = Z_0\frac{50+Z_c tnah(\gamma_m L)}{Z_c+50tanh(\gamma_m L)}$ is the input impedance of the transmission line, $\gamma_m = \alpha_m + j\frac{2\pi f}{c_0}n_m$ is the RF propagation constant, $F(u) = \frac{1-\exp(u)}{u}$, and $u_\pm = \pm\alpha_m L + j\frac{2\pi f}{c_0}(\pm n_m - n_o)L$. In our simulation, we calculate EO_{S21} at the given electrodes thickness of 0.9 μm. The width of the signal strips are optimized to match 50-ohm load and generator and to minimize the mismatch between n_m and optical group index (n_o). The simulated BW/ V_π is shown in Supplementary Figure 5**d**. It shows the largest BW/ V_π value at w= 4 μm and the optimized electrodes gap is 7 μm. We adopt this design in our experiments and the properties of the transmission line is shown in Supplementary Figure 5**a-c** (purple lines). Supplementary Figure 5**a** shows RF losses of the transmission line, α_m = 5.94 dB/cm at 80 GHz. The index mismatch is below 5.6 % in the frequency range from 1 GHz to 120 GHz (Supplementary Figure 5**b**). The mismatch of $|n_m - n_o|$ and Z_c can be further reduced by adjusting the thickness of the SiO_2 (h_{SiO}) and the width of signal strip (w_s), respectively, without compromising the V_π simultaneously. The RF properties of a further optimised structure (w_s= 17 μm and h_{SiO} = 2.5 μm) is shown in Supplementary Figure 5**a-c** (yellow lines). A 1-cm device with the optimised structure gives a V_π of 2.65 V according to a simulated $V_\pi L$ of 2.65 Vcm, and a 3-dB EO bandwidth of 110 GHz. The V_π value can be further reduced to 2 V and achieve a 70-GHz 3-dB bandwidth in a 13.25-cm modulator. (Supplementary Figure 5**e**)

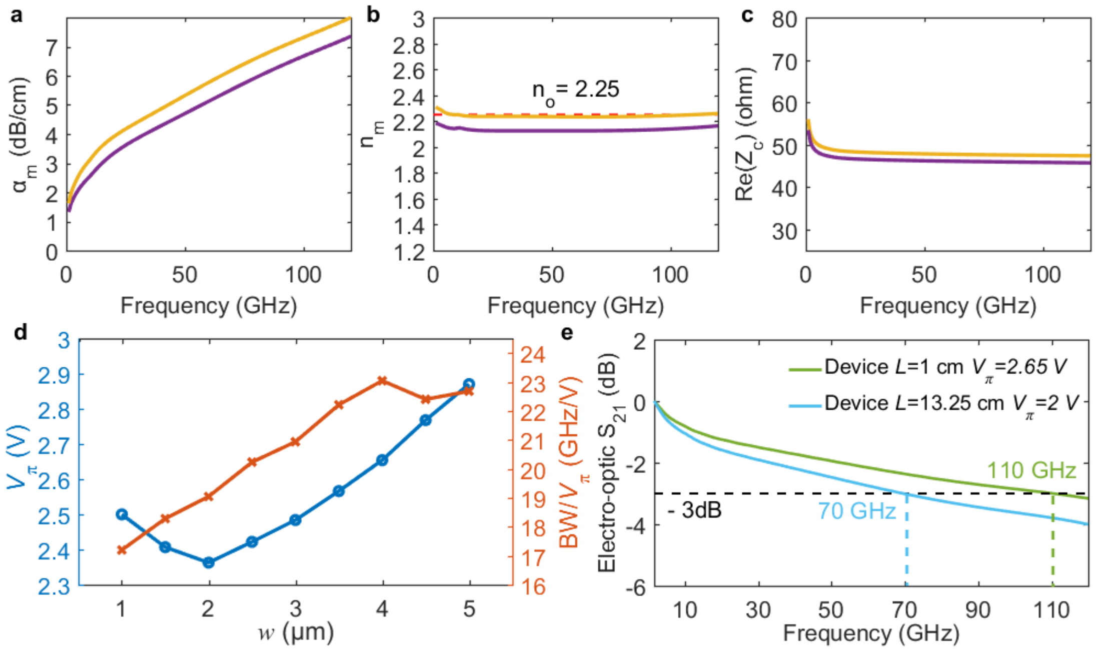

Supplementary Figure 5| Design of travelling wave electrodes and energy efficiency. a RF attenuation, **b** RF effective mode index, **c** characteristic impedance Z_c of the present modulator with w_s= 23.6 μm and h_{SiO}= 4.7 μm (purple lines) and w_s= 17 μm and h_{SiO}= 2.5 μm (yellow lines). **d** V_π and BW/V_π as a function of the width of the LN waveguides. **e** Overall EO modulation response of the optimized design (w_s= 17 μm and h_{SiO}= 2.5 μm). Green line is for 1-cm long modulation length and blue is for 13.25 cm and the estimated V_π values are 2.65 V and 2 V, respectively. The calculated 3-dB EO bandwidth is 110 GHz and 70 GHz, respectively.

Supplementary Note 5 | IQ modulator with EO phase shifters

To further compare the devices with TO phase shifter and EO phase shifter, we fabricated an LNOI-based IQ modulator with EO phase shifters for DC bias voltage control (Supplementary Figure 6**a**). In Supplementary Figure 6**b**, the 3-mm-long EO phase shifter was used to introduce a 90° phase difference between the In-phase/Quadrature branches. It takes about 5V to set the modulator to the optimum modulation bias point. We use EO phase shifters to adjust the IQ modulator operating at the optimum bias points to generate 64 Gbaud QPSK signals. We can observe that constellation diagram of the QPSK signals became seriously degrade and the BERs deteriorate from 8.6×10^{-5} to 7.4×10^{-3} in 5 minutes (Supplementary Figure 6 **c-d**). It turns out that the EO effect can be used to control the LNOI modulator but we need to use bias control board to maintain a stable operation.

Supplementary Table 1 compares several performance metrics of the demonstrated IQ modulators with TO and EO phase shifters. The modulators show no difference in the value of V_π and EO bandwidth because of the same travelling-wave electrodes and optical waveguides design. The TO phase shifter generates extra power consumption compared to the EO phase shifter, but the size is much compact and the bias control is more stabilized.

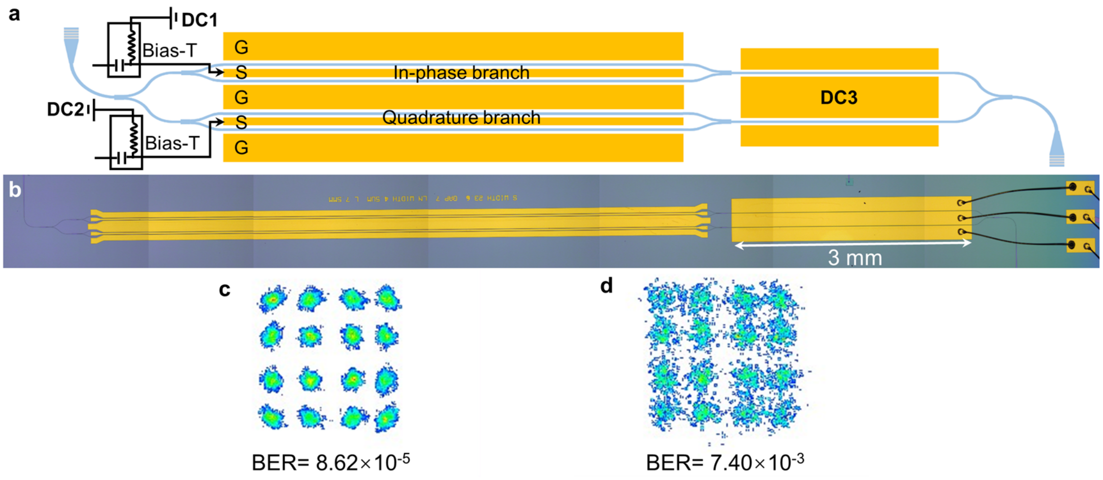

Supplementary Figure 6| IQ modulator with EO phase shifters. a Schematic of an integrated LN IQ modulator with EO phase shifters for DC bias voltage control. **b** Microscope image of a fabricated device. **c, d** Measured constellation diagrams for 64 Gbaud 16 QAM signals under optimum bias points **c** at once and **d** 5 minutes later.

Supplementary Table 1. Comparison of several performance metrics of the 7.5-mm IQ modulators with TO and EO phase shifters

	V_π (V)	3-dB EO bandwidth (GHz)	Length of phase shifter (mm)	Power consumption caused by phase shifters (mW)	Whether the bias control is stable
IQ modulator with TO phase shifters	3.1	> 67	0.16	~16.6	Yes
IQ modulator with EO phase shifters	3.1	> 67	3	0	No

Supplementary Note 6| Modulator energy considerations

We can further reduce the energy consumption of the LNOI-based IQ modulator through reducing the V_π .We fabricated a MZM with a long modulation length of 18 mm. In Supplementary Figure 7, it demonstrates a record low V_π of 1.25 V and high EO bandwidth of 43 GHz.

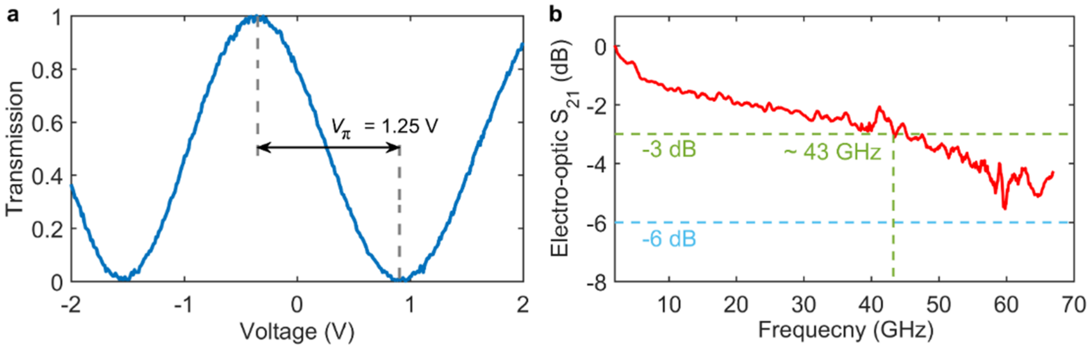

Supplementary Figure 7| Characteristics of an 18-mm-long LNOI-based MZM. **a** Normalised optical transmission as a function of the applied voltage, showing V_π of 1.25 V. **b** Small signal response EO bandwidths (S_{21} parameter) of the 18-mm Mach-Zehnder modulator.

Supplementary References

[S1] Ghione, G. *Semiconductor devices for high-speed optoelectronics*. (Oxford Univ. Press, Oxford, 2009).
